# Ecological risk and source analysis of soil heavy metals pollution in the river irrigation area from Baoji, China

**DOI:** 10.1371/journal.pone.0253294

**Published:** 2021-08-02

**Authors:** Jun Zhang, Yu Gao, Ningning Yang, Enhua Dai, Minghang Yang, Zhoufeng Wang, Yani Geng

**Affiliations:** 1 Shaanxi Key Laboratory of Disaster Monitoring and Mechanism Simulation, Baoji University of Arts and Sciences, Baoji, China; 2 Key Laboratory of Subsurface Hydrology and Ecological Effect in Arid Region of Ministry of Education, Chang’an University, Xi’an, China; Al Mansour University College-Baghdad-Iraq, IRAQ

## Abstract

Due to various human activities, soil quality under different land use patterns is deteriorating all over the world. This deterioration is very complex in the river irrigation area and is caused by multi-point and non-point source pollution and seasonal variation. Therefore, the characteristics and sources of soil metal pollution in river irrigation area of Baoji city were analyzed. The contents of 8 metals were given by ICP-MS, in the soil samples. Statistical methods, geo-accumulation index (*I*_geo_) and potential ecological risk index (*RI*) were conducted to evaluate the spatial distribution features, sources and ecological risks of metal contamination from the study area soil. Principal component analysis and cluster analysis were used to analyze the pollution sources of metal. The analysis showed that Cd is the most polluted, and human activities represented a great impact on the contents of Zn, Ni, Cu and Cd in soil, Cd post moderate-strong pollution and strong risk, Cd has a maximum Igeo value of 3.17. All rivers were at risk of moderate pollution levels in study. Among them, some rivers had even reached strong pollution level. Pollution caused by human activities was the most significant pollution source of metal in the research area soil.

## Introduction

Soil heavy metal pollution f tensive development of industry, agriculture and transportation may lead to the overall impact of heavy metals on urban ecosystems [1–5]. Heavy metals are highly toxic, not easily biodegradable and easy to be enriched by organisms. They can be transferred into human body or other organisms through the food chain, which pose direct or indirect threats to human health [6, 7]. The study of heavy metal pollution is especially important in China, where the economy is developing rapidly [8, 9].

At present, many studies focus on the content and morphology of heavy metals in environmental media such as soil, water, plants and river sediments, as well as the assessment of heavy metal pollution risks and pollution sources [10–14]. There is little analysis on the spatial distribution of heavy metal pollution in the soil surrounding urban river irrigation channels. The research on soil heavy metal pollution in Baoji mainly focuses on farmland soil, coal mine soil and surrounding soil of urban sewage discharge area [15–19]. Their research showed that heavy metal pollution in the natural environment of Baoji city has emerged. However, the systematic study and source analysis of heavy metal pollution in the surrounding soil of major river irrigation channels in cities are less. Rapid urbanization has led to a decline in the quality of river irrigation due to the massive discharge of sewage, which brings potential heavy metal pollution to the soil due to agricultural irrigation in times of drought. Weihe irrigation area is located in the river irrigation area in the west of Guanzhong, Shaanxi province. This area is the largest grain and oil production base in the province, and the largest irrigation area in Guanzhong plain.

Considering the risks posed by soil metals surrounding river irrigation channels, a research was conducted to analyze the heavy metal contamination status and sources of heavy metals from the surrounding soil of Weihe river in Baoji. This area belongs to Baojixia irrigation area. Baojixia irrigated area is one of the top ten irrigation districts in China. The main water source for irrigation is runoff from Weihe river, supplemented by groundwater. The irrigated area is 194,400 hm^2^, and the agricultural population in the irrigated area is about 2.07 million. It is the largest grain, vegetables, oil, fruit production base in Shaanxi province, known as the "first granary" of Shaanxi province. The research objectives were as follows: (1) to determine the contents of 8 metals and analyze the spatial distribution features of soil metal contamination; (2) to assess the risk of soil pollution by using geo-accumulation index method (*I*_*geo*_) and potential ecological risk index method (*RI*); (3) to apportion sources of heavy metals using the multivariate statistical method.

## Materials and methods

### Study area

Baoji is located in the Baojixia irrigated area in the west of Guanzhong. The Weihe river runs through the area, with a total area of 1.8 million hm^2^ and a total population of 3.72 million. In 2015, the cultivated land area was 146,000 hm^2^, accounting for 50.3% of the cultivated land area commonly used in Shaanxi province. The effective irrigated area is 90,000 hm^2^, accounting for 72.9% of the total irrigated area in the whole basin. The study area is located in Longhai, Baocheng, Baozhong railway interchange. It is the sub-central city of Guantian Economic Zone and the second largest city of Shaanxi province. Industrial enterprises such as machine tool factory, energy processing enterprise, new material research and development base, titanium processing enterprise and so on are densely distributed in the urban area. It is also a typical valley industrial city [19]. Based on field investigation, it was found that there are a large number of factories and mechanical processing plants around each river, among which there are sand mining fields around Qianhe River, and the waste and waste water were directly discharged into the river.

**Fig 1:**
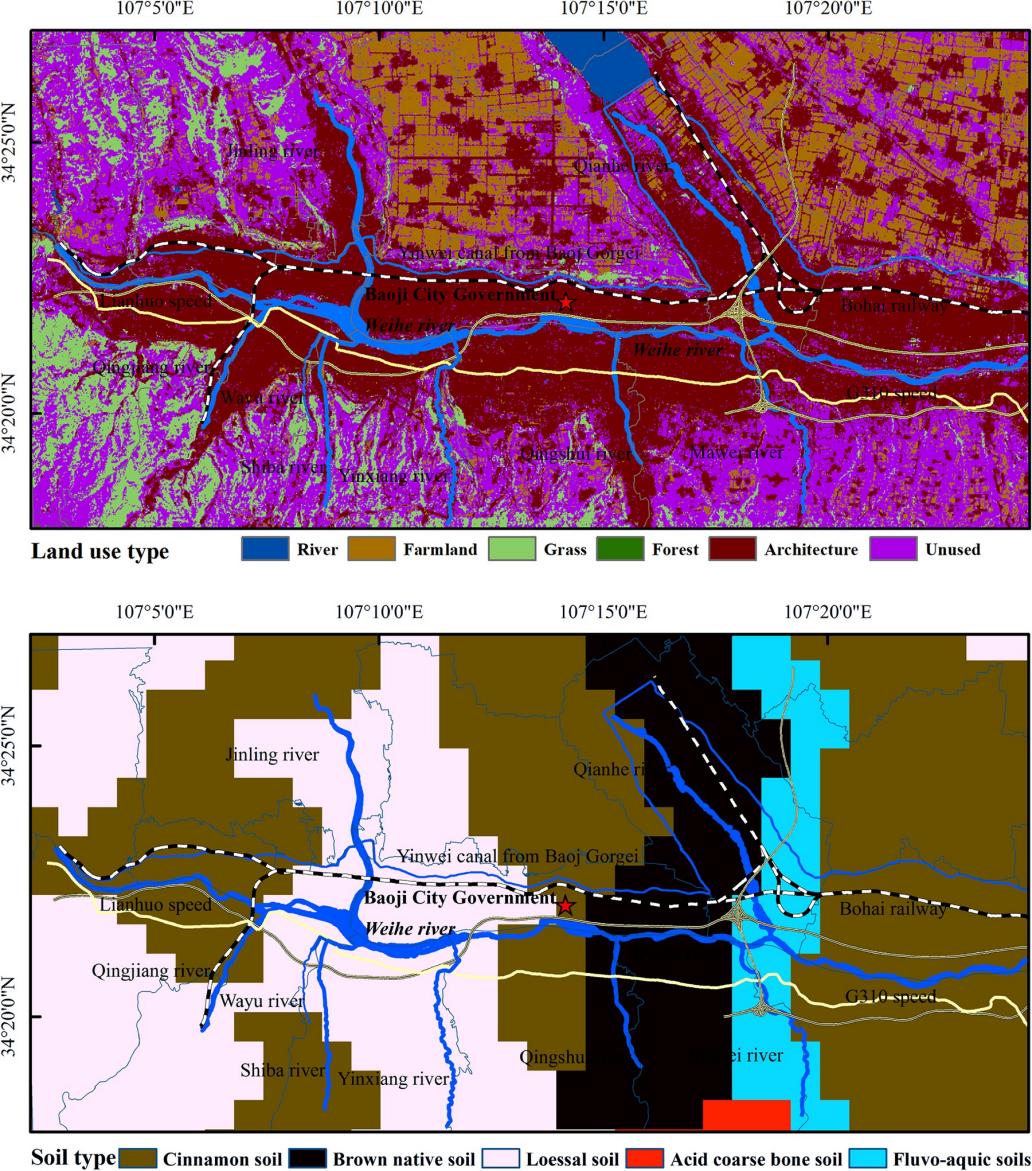
Soil type and land use type in the study area.

**Fig 2:**
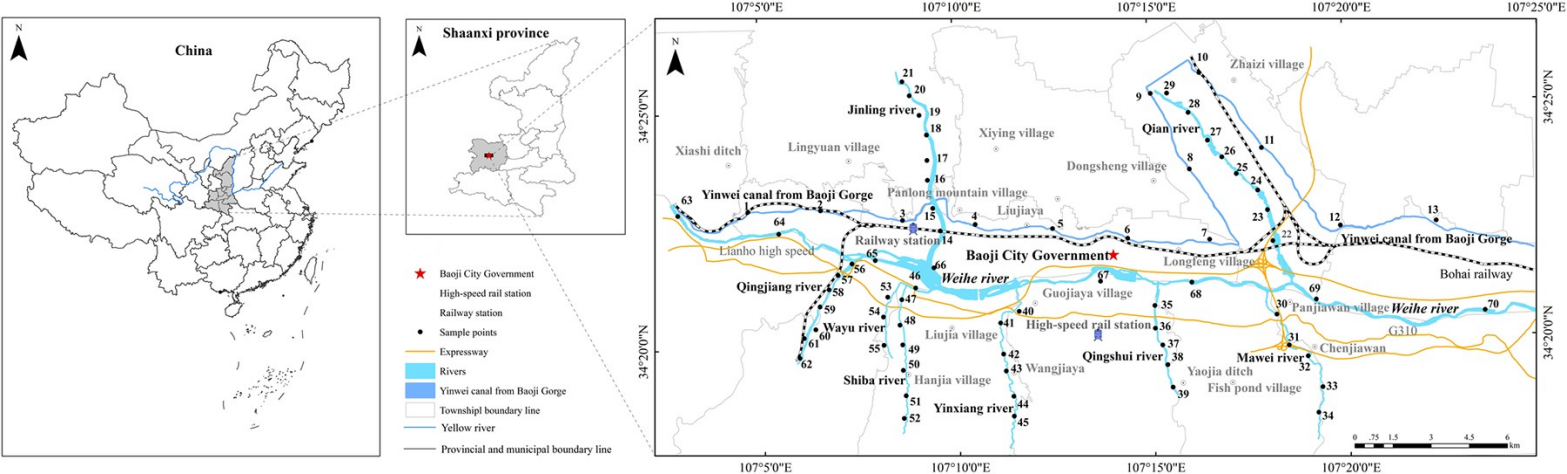
Sampling point layout.

The study area is located between 107°0′30′′~107°26′4′′ E and 34°15′10′′~34°29′6′′ N, with a warm temperate sub-humid climate, cold and dry in winter, warm and rainy in summer, with an average annual rainfall of about 600 mm. The river irrigation channels in the study area include Weihe river, Qingjiang river, Jinling river, Qian river, Yinwei canal, Wayu river, Shiba river and Yinxiang river [20, 21]. The soil types in the study area mainly include cinnamon soil, brown native soil, loessal soil, acid coarse bone soil and fluvo-aquic soil. The main types of land use in the study area are architecture, farmland, river, grass, forest and unused (see Fig 1). According to the field survey, the digital elevation model (DEM) and GIS technology were combined to distribute points along the river. The sampling points were all within 100 meters from the river, and the sampling points were spaced 1–2 km apart by river length. A total of 70 samples of surface soil 0~20 cm were collected according to relevant literature [22–24], the sampling points were arranged as shown in Fig 2. The sampling was located by the handheld GPS recorder, and samples were collected at the sampling point in the shape of a 5 m × 5 m area in the shape of a plum-shaped shape. Each mixed sample was about 1.0 kg, packed into a polyethylene sealed bag and brought back to the laboratory for testing.

### Sampling and laboratory analysis

The soil samples were stripped of stone, roots and other impurities, dried naturally, ground and passed through a 100-mesh nylon sieve before being stored in a sealed bag for future use. The mean values of physicochemical properties of soil samples measured according to the method of soil agrochemical analysis [25] were shown in Table 1. Soil sample 0.1000g was placed in the digestion tank, and a little ultra-pure water was added to moisten it. Then HNO_3_-HF-HClO_4_ (1:2:2) triacid system was also added into the digestion tank [26]. Digestion was conducted in a fully automatic digestion machine (Deena Ⅱ, Thomas Cain, US) and heated up to 180°C to dissolve samples into a colorless and odorless solution. Then the solution was taken off and cooled to keep in a constant volume 50 mL volumetric bottle. After filtered with 0.45m filter membrane, the product was transferred to 10 mL centrifugal tube for testing. After digestion, Cr, Mn, Ni, Cu, Zn, As, Cd and Pb were determined in soil samples by ICP-MS (Nexion 350x, PE, US). In the experiment, Chinese national standard soil samples (GSS-25) were used for quality control, and the element recovery rate was controlled between 92 and 105%. Each sample was tested three times, with a blank set, with a relative standard deviation (RSD) of less than 10%. All the reagents are super pure, and the water is super pure.

**Table 1:** The physical chemical properties of the soil.

Total nitrogen (TN) /g·kg^-1^	Total phosphorus (TP) / g·kg^-1^	Organic matter (OM) / g·kg^-1^	pH	Cation exchange capacity (CEC) /cmol·kg^-1^
1.78	2.53	31.5	8.31	8.90

### Evaluation model and method

In this study, geo-accumulation index method (*I*_*geo*_) [27, 28] and potential ecological risk index method (*RI*) [29, 30] were used to evaluate the risk of soil pollution.

#### Geo-accumulation index method

*I*_*geo*_ can quantitatively reflect the degree of heavy metal pollution in the soil, as shown in formula (1):

$$I_{\mathrm{geo}}=\log_{2}[C_{n}/KB_{n}]$$ (1)

where, *C*_*n*_ is measured value of heavy metal content, *B*_*n*_ is the geochemical background value of element n measured, and *K* is a constant, which is a correction to the change of background value that may be caused by diagenesis (generally *K* = 1.5). See Table 2 for the classification criteria of *I*_*geo*_ [31].

**Table 2:** *I*_geo_ classification standards.

Classification level	Geo-accumulation index	Pollution level
**1**	*I*_geo_≤0	No pollution
**2**	0< *I*_geo_≤1	Light—moderate pollution
**3**	1< *I*_geo_≤2	Moderate pollution
**4**	2< *I*_geo_≤3	Medium-strong pollution
**5**	3< *I*_geo_≤4	Strong pollution
**6**	4< *I*_geo_≤5	Strong-severe polluted
**7**	5< *I*_geo_≤10	Severe pollution

#### Potential ecological risk index method

*RI* [29] was defined by Hacanson (Swedish) to explain the potential ecological risk of heavy metal pollution on soil. It takes into account not only the concentration of multiple elements and toxicity levels, but also ecological sensitivity and synergistic effects. The calculation process is shown in formula (2)—(4).

$$C_{f}^{i}=\frac{C_{i}}{C_{n}^{i}}$$ (2)

$$E_{r}^{i}=T_{r}^{i}\times C_{f}^{i}$$ (3)

$$RI=\sum_{i=1}^{m}E_{r}^{i}$$ (4)

where, *C*^*i*^_*f*_ is the single pollution coefficient, *C*_*i*_ is the measured value of the element, *C*^*i*^_*n*_ is the standard reference ratio (the background value of soil in Shaanxi province is taken as the reference ratio) [32], *E*^*i*^_*r*_ is RI of element *i*, *T*^*i*^_*r*_ is the toxicity coefficient of element *i*. According to relevant literature [30, 33, 34], the toxicity coefficients of 8 heavy metals in this study are Cr (2), Mn (1), Ni (5), Cu (5), Zn (1), As (10), Cd (30) and Pb (5). The potential ecological risk rating criteria of soil are shown in Table 3 [30, 33, 34].

**Table 3:** *E_*r*_^*i*^* and *RI* classification standards.

*RI* of element *i*, *E*_*r*_^*i*^	Level of ecological hazard	*RI*	Ecological risk levels
***E***^***i***^_***r***_ **< 40**	Slight	***RI* < 150**	Slight
**40 ≤ *E***^***i***^_***r***_ **<80**	Moderate	**150 ≤ *RI* < 300**	Moderate
**80 ≤ *E***^***i***^_***r***_ **<160**	Strong	**300 ≤ *RI* < 600**	Strong
**160 ≤ *E***^***i***^_***r***_ **<320**	Stronger	***RI* ≥ 600**	Stronger
***E***^***i***^_***r***_ **≥ 320**	Extremely intense	-	-

### Data processing method

In this manuscript, Excel 2010 was used for data processing, SPSS 19.0 was applied for clustering analysis and principal component analysis (PCA), and ArcGIS 10.5 was conducted to make the spatial distribution map of heavy metal elements.

## Results and discussion

### Heavy metal content of soil in the study area

The soil around the river irrigation canal in Baoji city has been polluted by some heavy metals (Table 4). The average concentration of Cd was higher than the limit of chinese national soil environmental quality standard [35], and the coefficient of variation was 120%. The average contents of Cd, Zn and As were 1.14 mg·kg^-1^, 269.02 mg·kg^-1^ and 18.41 mg·kg^-1^, were 12.13 times, 3.88 times and 1.64 times of the background values in Shaanxi province, respectively. The exceeding rates of Cd and Zn were 68.57% and 27.14%, respectively. It indicates that these three heavy metals are the main pollutants. And the contents of other heavy metal elements did not exceed the background values in Shaanxi province. In addition, the maximum values of Mn, Ni, Cu, As, Cd and Pb all exceeded the background values in Shaanxi province. Indicating that they accumulated significantly in the soil around the river irrigation canal in Baoji city. The variation coefficient can reflect the average variation degree of each sampling point, and also reflect the influence degree of the heavy metal element by human activities. The variation coefficients of Ni, Cu, Zn and Cd were all above 36%, showing strong variability [36], indicating that human activities represented a large impact. Besides, the spatial distribution was uneven, indicates that the source of the pollution is complex [31, 37, 38]. In the previous research results of zhang jun and yi wenli et al., the mean Cd content was 0.77 mg·kg^-1^ and 0.76 mg·kg^-1^, which were 8.2 and 8.04 times of the soil background value in Shaanxi province, respectively, both lower than the results of this study. The reason may be that there was a large amount of agricultural land around the river irrigation channels in the study area. During the drought, river water was used to irrigating the soil by farmers, which may cause the potential risk of heavy metal pollution to the local soil. Moreover, excessive use of pesticides and fertilizers also led to increased heavy metal pollution. As being thought to come mainly from agricultural pollution. In general, in study areas with high heavy metal content in the soil, the surrounding heavy industry enterprises are densely distributed and the transportation is developed, which increased the accumulation of Zn and Cd [39–41], and low heavy metal contents are mainly residential areas and new energy industries. Therefore, it can be considered that industrial pollution discharge is also an important source of heavy metal pollution in the soil surrounding the study area. Table 4 is descriptive statistics of heavy metal content in the soil of the study area, The "-" in the table indicates that it was not detected.

**Table 4:** Heavy metal contents in soil of study area (n = 70).

Elements	The minimum value	The maximum value	The median	The average value	Standard deviation	Coefficient of variation	Background value of Shaanxi province	Soil environmental quality standard(GB15618-2018)	Excess rate
	(mg·kg^-1^)					%	(mg·kg^-1^)		%
**Cr**	27.75	41.23	34.02	33.75	2.77	8	62.5	250	-
**Mn**	23.80	64.46	43.33	43.10	8.65	20	557	-	-
**Ni**	4.34	69.44	6.75	8.92	8.61	97	28.8	190	-
**Cu**	3.08	101.57	10.24	14.17	12.60	89	21.4	100	1.42
**Zn**	138.32	818.61	244.70	269.02	109.81	41	69.4	300	27.14
**As**	14.41	21.32	17.82	18.41	1.56	9	11.2	25	-
**Cd**	0.31	9.36	0.72	1.14	1.36	120	0.094	0.6	68.57
**Pb**	0.75	38.10	4.51	5.32	4.43	83	21.4	170	-

### Spatial distribution characteristics of heavy metal content in soil

**Fig 3:**
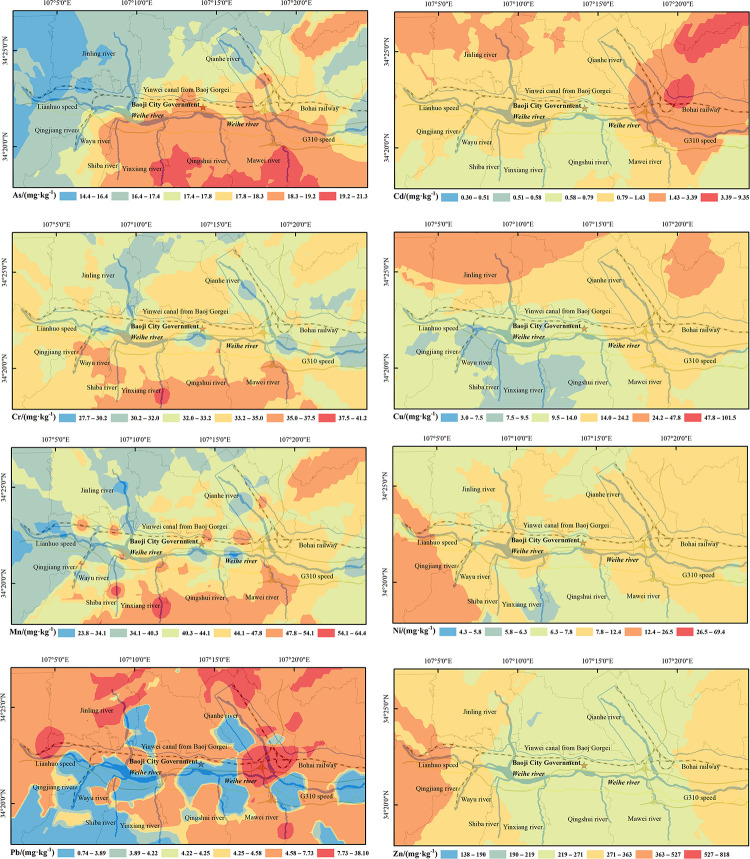
Distribution maps of heavy metal content in soil around the river in Baoji City.

The spatial distribution of soil heavy metal content in the study area was shown in Fig 3. It can be seen that the high values of Cr, Mn, As and Zn in the contents of 8 heavy metals all occurred on the south bank of the Weihe river, such as high values of Cr in Wayu river, Shiba river, Yinxiang river and Mawei river. High values of Mn in Shiba river and Yinxiang river, while other high Mn areas were patchy on both sides of Weihe River. As high value in Yinxiang river, Qingshui river and Mawei river, while the other high values were mainly distributed in the southeast of Weihe River. The content and variation coefficient of Cr, Mn and As were relatively low. Combined with soil types and land use types, it was found that the soil types in the high-value area were mainly cinnamon soil and brown native soil, while the land use types were mainly unused land, forest and grass, which may be affected by natural factors. The highest Cd values were mainly found in the northeast of Qian river and Yinwei canal, and the northwest of Jinling river. Roads are densely distributed in the northwest of Jinling River, where 212 provincial road, Xiangong passenger station of Chencang district and Qipanshan cemetery reception meet. The high value area in the northeast of Yinwei canal was the intersection of National highway 344, Yinkun highway and Xunfeng highway. Bohai railway line, Baolin railway and Fengxiang west railway station converge here. In addition, this area belongs to Fengxiang county, the largest county in Baoji, with a large population and developed road traffic. Pb high value zones were mainly located at the junction of Qian river and Weihe River, and in the north of Jinling River and Qian River. Other high value zones were located in the north of Weihe river, which was close to the high value zone of Cd. It is found that the dense roads near the high value areas of Cd and Pb may be attributed to the influence of Shaanxi Power Company, railway, Baoji Concrete Company, intersection of expressway and railway. The high values of Zn were mainly distributed in the west of Qingjiang river and the northwest of Weihe river. There were many factories in this area, including Baoji Gaojia Galvanizing Plant and Baoji Qinchuan Air Engine Factory. High value areas of Cu were mainly located in the northwest of Jinling river and the northeast of Yinwei canal. The high value areas in the northwest of Jinling river are surrounded by factories, including baoji machine tool Supporting Industrial Park, Baoji Tiandiao Chemical Company, Baoji Guanghuan Machine Tool, Baoji Chengyue Hongrui Machinery Company and other factories. There are also many factories near the high-value area in the northeast of Yinwei canal, including Baoji Zhengyuan Chemical, Baoji Hengxing Petrochemical, Changqing Energy chemical and other factories. High value areas of Ni were mainly distributed in the southwest of Qingjiang river and Wuayu river. There are a large number of factories in this area, including Baoji Shuntong Tire factory, Taiping Auto Parts company, Fuchang Cement Factory, Baoji People’s Flour mill, Yaste sofa bed and other factories. The content in the rest areas was relatively small. It is found that there are a large number of heavy metal processing plants, auto parts companies and energy companies in the vicinity of the high content zones of Zn, Cu and Ni, which may be attributed to the influence of factory production. The areas with low content of heavy metals were less affected by human activities and had little influence on the import of heavy metals into soil environment.

### Analysis of evaluation results

#### The evaluation result of *I*_*geo*_

**Fig 4:**
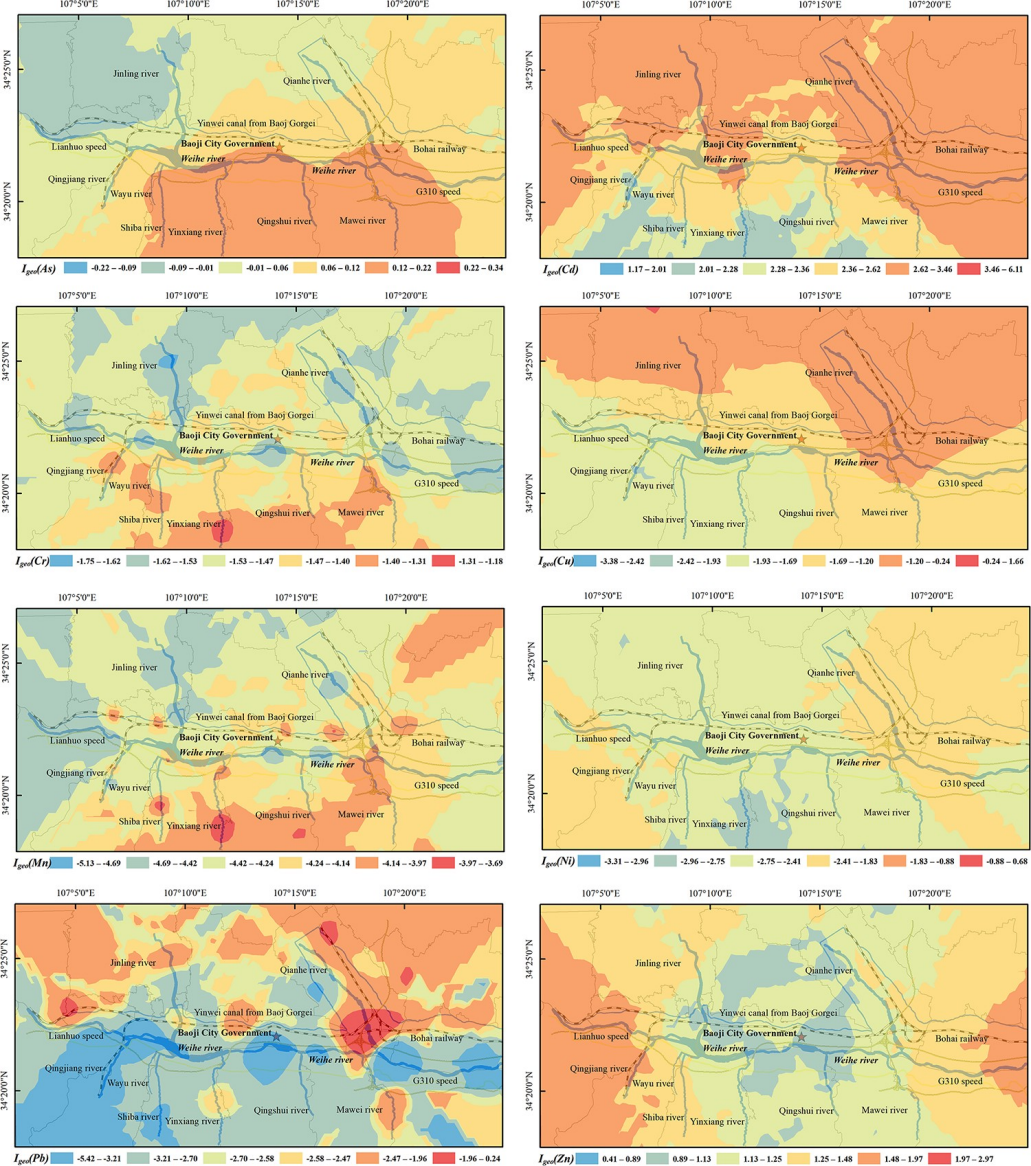
The spatial distribution of soil heavy metal *I*_*geo*_ in the study area.

Evaluation results of *I*_*geo*_ were revealed in Fig 4. Cr, Mn, Ni, Cu and Pb were no pollution in the soil. As was light-moderate pollution, Zn was moderate pollution, and Cd was moderate-strong pollution. The *I*_*geo*_*s* of 8 heavy metals in the whole study area were exhibited the order Cd > Zn > As > Cr > Cu > Ni > Pb > Mn, in which Cd and Zn were greater than 1, As was between 0 and 1, and the *I*_*geo*_*s* of other heavy metals were all negative. In addition, As contributed the most pollution to the soil around Mawei river and Yinxiang river, which is light-moderate pollution, and there are few human activities in this area. Cd and Pb are the main pollutants in the soil around the Yinwei canal, Jinling river and Qian river, the area has developed traffic and dense roads. Zn is the major pollutant in the soil around Qingjiang river, Weihe river and Yinwei canal. The pollution index of most sampling sites is greater than 1, which is a moderate pollution with many factories in the high-value area. The *I*_*geo*_ maxima of Ni and Cu were all revealed in the soil along the Qian river, indicating that Ni and Cu were higher in the soil around the Qian river, which was related to the metallurgical plants, mines, power plants and urban industries along the Qian river.

#### Assessment results of potential ecological risk

**Fig 5:**
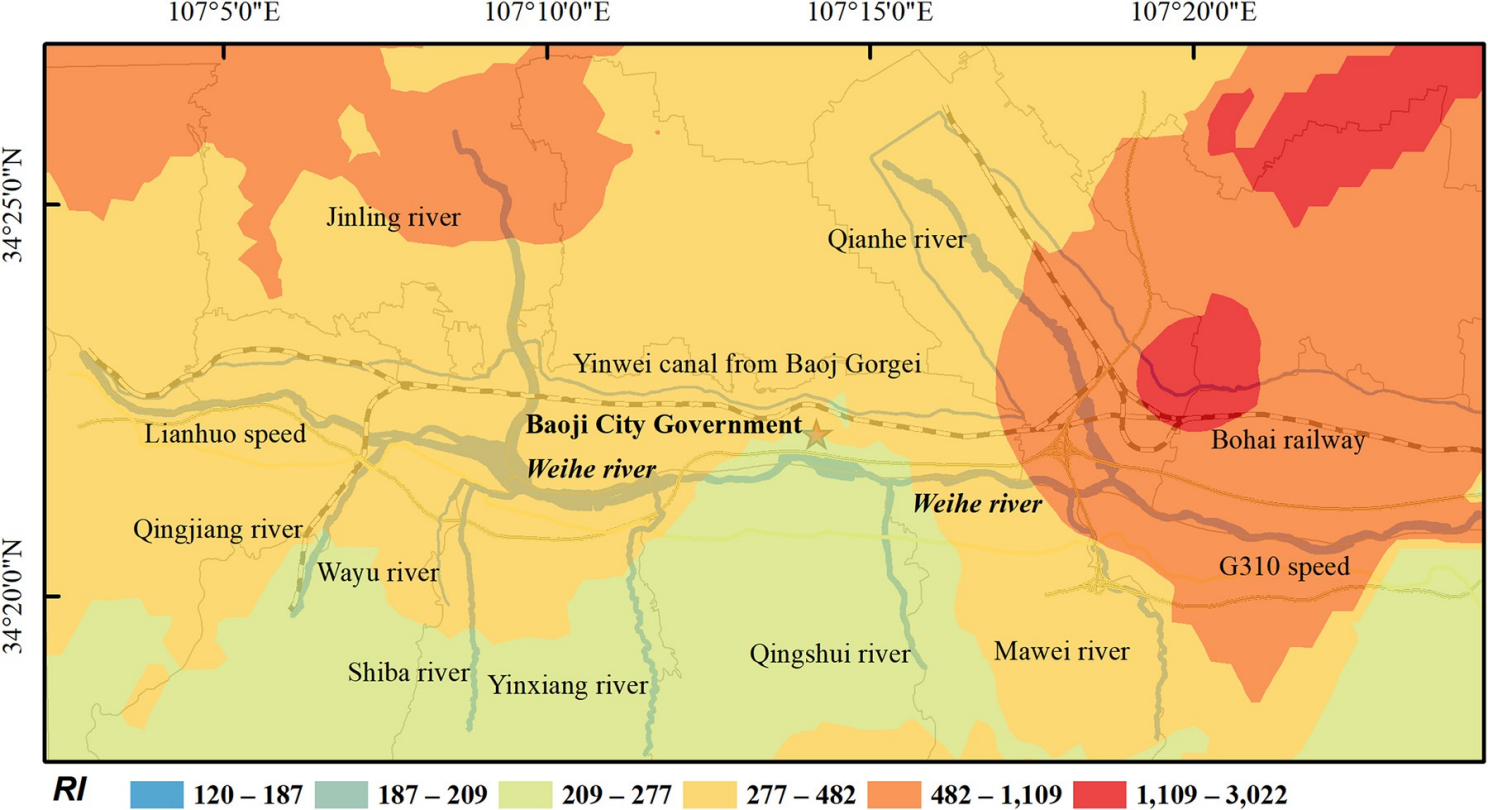
*RI* of heavy metal in soil in the study area.

The highest mean *RI* of 8 heavy metals was Cd, followed by As, Zn, Cu, Ni, Pb, Cr, Mn (Fig 5). As can be seen from *E*_*r*_^*i*^ of heavy metals, Cd in the study area presented a strong risk (*E*_*r*_^*i*^ = 318.14) and was the main pollutant. The *E*_*r*_^*i*^ maximum values of Mn, Ni, Cu, Zn and Pb were found in the banks of Yinxiang river, Qingjiang river, Jinling river and Qian river, respectively, and all of them were slightly polluted. The maximum value of Cd was found along the Yinwei canal, reaching extremely high pollution level. The *RI* values of heavy metals in river soil exhibited the order Yinwei canal (593.15) > Qian river (492.07) > Jinling river (481.67) > Shiba river (472.86) > Mawei river (341.18) > Qingjiang river (297.72) > Yinxiang river (237.57) > Weihe river (235.24) > Qingshui river (222.31) > Wayu river (202.86), all rivers were at risk of moderate pollution levels. Among them, the soil along Yinwei canal, Qian river, Jinling river and Mawei river has even reached strong pollution level. The field investigation shows that the dense roads and numerous factories near the area may be the main cause of pollution in the area. The overall *RI* of the study area was 345.23, showing a strong risk level, and it is suggested that relevant departments should attach great importance to it.

### Source analysis of soil heavy metals in the study area

**Fig 6:**
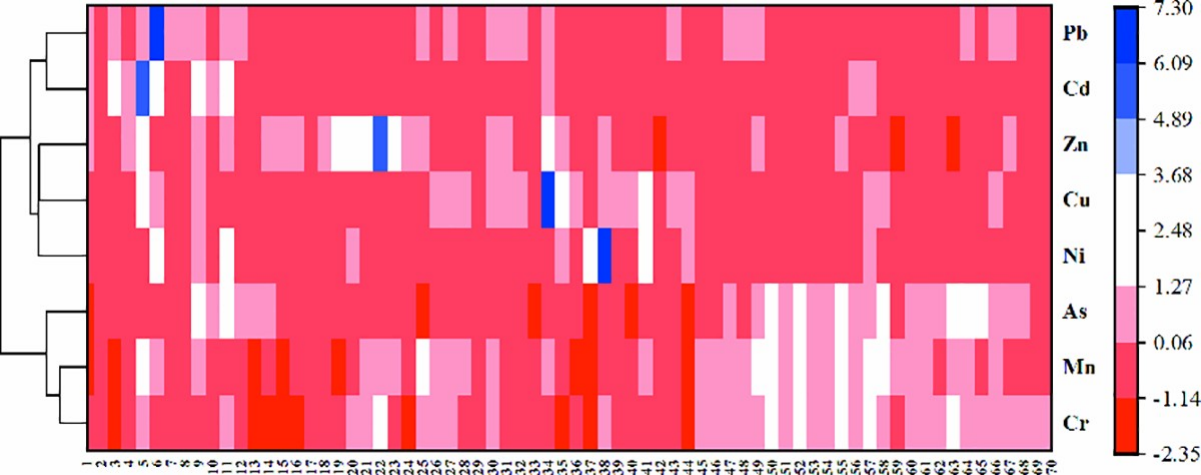
Cluster analysis heat map based on ward method.

In this manuscript, the cluster analysis and PCA were applied to analyze the sources of Cr, Ni, Mn, Cu, Pb, Cd, Zn and As in the soil from the study area. The Euclidean distance was conducted to draw the clustering tree of 8 heavy metal elements based on the Ward method (Fig 6). The cluster diagram can vividly reveal the relationship between various heavy metals and more intuitively obtain the pollution source of heavy metals. Based on Fig 6 and actual environmental factors, heavy metals can be divided into three components, Cd—Pb, Cr—Mn—As and Zn—Ni—Cu, indicating that each component may come from different pollution sources in the soil.

The results of PCA (Table 5) showed that there are three eigenvalues greater than 1, namely 2.18, 1.49 and 1.41, so three principal components can be obtained. The loading of Cr, Mn and As in component 1 was higher, and the variance contribution rate was 27.29%. The loading of Cd and Pb in component 2 was higher, and the contribution rate was 18.66%. Zn, Ni and Cu had higher loading in component 3, and the contribution rate was 17.66%. Indicating that the pollution sources of each component are different, which is consistent with the results of cluster analysis. Compared with clustering analysis and PCA, the soil heavy metal pollution sources in the study area were divided into three categories, the first kind of source was contamination of natural soil parent material, 27.29%, the second source was transportation pollution, at 18.66%, the third was industrial pollution sources, at 17.66%, and three kinds of sources were cumulative contribution rate of 63.62%.

**Table 5:** PCA results of 8 heavy metals in soil.

Component	Initial Eigenvalues			Rotation eigenvalues			Heavy metals	Rotating ingredient matrix		
	Eigenvalues	Variance contribution rate /%	Cumulative contribution rate /%	Eigenvalues	Variance contribution rate /%	Cumulative contribution rate /%		1	2	3
**1**	2.18	27.34	27.34	2.18	27.29	27.29	Cr	0.89	-0.04	0.03
**2**	1.76	22.01	49.36	1.49	18.66	45.96	Mn	0.88	0.07	0.06
**3**	1.14	14.26	63.62	1.41	17.66	63.62	Ni	-0.05	0.10	0.60
**4**	0.85	10.70	74.33	-	-	-	Cu	-0.02	0.35	0.55
**5**	0.78	9.76	84.09	-	-	-	Zn	0.03	-0.18	0.80
**6**	0.62	7.80	91.90	-	-	-	As	0.77	-0.00	-0.16
**7**	0.39	4.98	96.89	-	-	-	Cd	0.03	0.76	0.25
**8**	0.24	3.10	100.00	-	-	-	Pb	0.00	0.86	-0.07

The PCA and cluster analysis are effective multivariate statistical methods to distinguish sources of heavy metals. The sources of heavy metals in soil are not only the natural causes of the parent soil, but also the human factors such as the distribution of industrial enterprises and the traffic network in the study area. The loading of Cr, Mn and As in component 1 was higher, indicating that these three elements in the soil mainly come from the parent soil. As can be seen from Table 4, the average values of Cr, Mn and As are lower than or close to the soil background value of Shaanxi Province, which are in the degree of no pollution. The variation coefficients of Cr, Mn and As are 8%, 20% and 9%, which are slight variations, indicating that they are less affected by human activities. Based on the distribution of heavy metal content in soil around rivers in Baoji city (Fig 3), the spatial distribution of Cr, Mn and As content are mainly in the southern part of the study area. The parent soil type is mainly cinnamon soil and tidal soil, while the land use types are mostly unused mountainous area. Therefore, it can be inferred that the first principal component is mainly from natural sources (parent soil).

The loading of Cd and Pb in component 2 was higher. As can be seen from soil heavy metal content in the study area (Table 4), the average content of Cd was higher than the background value of soil environment in Shaanxi province, and the coefficient of variation is large, reaching a high degree of variation of 120%, indicating that it is highly influenced by human activities. The mean content of Pd was smaller than the background value of soil environment in Shaanxi province, but the variation coefficient is high (83%), indicating that Pd is also highly correlated with human activities. Studies have shown that Cd and Pb were derived from automobile exhaust emissions and lead-cadmium batteries17. In Fig 2, the spatial distribution of the region with high Cd and Pb content is consistent with the traffic conditions in the study area. The 212 provincial highway, Xunfeng Expressway, Yinkun Expressway and Baohan Expressway converge in the research area. The Longhai railway and Baocheng Railway pass through the area. And the complexity of the urban traffic roads leaded to the accumulation of Cd and Pb in the soil, due to vehicle exhaust emissions and early gasoline use. Therefore, it is inferred that the second principal component mainly comes from traffic pollution sources.

Zn, Ni and Cu had higher loading in component 3. According to Table 4, the average value of Zn were higher than the background value of soil environment in Shaanxi Province, and the variation coefficients of Ni, Cu and Zn were relatively large, reaching a strong degree of variation, indicating that contents of Ni, Cu and Zn are affected by human activities. Research shows that Ni, Cu and Zn were mainly from smelting, electroplating and chemical production. According to cluster analysis results and field investigation, the high values of Zn were mainly distributed in the west of Qingjiang river and the northwest of Weihe River. There are many factories in this region, including zinc plating factories and wind turbine factories. High value areas of Cu were mainly distributed in the northwest of Jinling river and the northeast of Yinwei canal, including energy chemical industry, petrochemical smelting and other factories. The Ni high value areas were mainly distributed in the southwest of Qingjiang river and Wayu river, including Shuntong tire, auto parts company, cement plant, etc. It is found in the field investigation that there are a large number of heavy metal processing plants, automobile parts cities and energy companies in the vicinity of areas with high content of Zn, Cu and Ni, which may be greatly affected by the production of industrial and mining enterprises. Areas with less element content are mainly residential areas and urban agglomerations, which are less polluted by the environment. This is consistent with the research results of Li Jiao [42, 43]. It can be inferred that the third principal component mainly comes from industrial production pollution sources.

It can be seen from principal component analysis results of the content of eight heavy metals in the soil (Table 5) that natural sources (27.29%) are still the main sources of heavy metals in the soil in the study area, followed by transportation sources (18.66%) and industrial sources (17.66%), and industrial and traffic pollution are the main sources of Zn and Cd that exceed the standards. This is related to the positioning of the research area as an industrial city for many years. Although it has undergone transformation and development in recent years, the impact of industrial development since the founding of the People’s Republic of China still exists. The contribution rates of traffic sources and industrial sources are relatively prominent, which is mainly because Baoji is a hub city of railway and highway transportation. Baoji railway and Longhai railway intersect each other, and a number of expressways and provincial highways pass through the territory. Baoji city is a typical valley city, traffic and industrial pollution is more likely to accumulate in the soil, which will cause more serious urban soil heavy metal pollution.

## Conclusion

Research on soil heavy metals in the river irrigation area from Baoji of northwest China pollution showed that: (1) Heavy metals have posed pollution to the soil around the river irrigation area in the study area. And Cd, Zn and As are the main polluting elements, among which Cd is the most polluted. (2) The soil in the study area revealed strong ecological risk and some rivers have reached the strong pollution level, and some rivers represented strong pollution levels. (3) Heavy metal pollution in the study area was mainly caused by human activities, and pollution sources were divided into three categories, contamination of natural soil parent material, transportation pollution, and industrial pollution sources.
